# Supplementary material for: Protocol: what are the ethnic inequities in care outcomes related to haematological malignancies, treated with transplant/cellular therapies, in the UK? A systematic review
Source: BMJ Open. 2025 May 21;15(5):e099354. doi: 10.1136/bmjopen-2025-099354 (PMC12096984; doi:10.1136/bmjopen-2025-099354)
Supplement: online supplemental file 1 [file bmjopen-15-5-s001.docx]

| Supplementary Table 2. **Summary of search strategy.** Ovid Evidence-based Medicine Reviews (EBMR) was used to format the search, with the syntax reflecting that used in EBMR*. | | |
| --- | --- | --- |
| Query | Description | |
| 1a | ((blood or h?em or liquid or myelo*) adj (cancer? or malignanc* or neoplasm? or tumo?r?)) | *Disease terms* |
| 2a | leuk?emia? |  |
| 3a | lymphoma? |  |
| 4a | 1a or 2a or 3a |  |
| 1b | (leuk?emia? adj (  (acute adj (myeloid or lymphoblastic or lymphocytic))  or  (chronic adj (myeloid or lymphocytic)) or  prolymphocytic or  (plasma adj cell)  )) |  |
|  | (lymphoma? adj (  hodgkins or  (((highgrade or (high adj grade)) or aggressive) adj/2 (bcell? or (b adj cell?))) or  burkitt or  (  large adj (bcell? or (b adj cell?)) adj (diffuse or (primary adj mediastinal))  ) or  follicular or  (  (tcell? or (t adj cell?)) adj peripheral  ) or  (mantle adj cell?) or  lymphoblastic or  (anaplastic adj ((large adj cell?) or largecell?)) or  (  primary adj ((central adj nervous adj system) or CNS)  ) or  lymphoplasmacytic or  (small adj lymphocytic)  ) or  (nodular adj lymphocyte adj predominant adj2 lymphoma?)) |  |
| 2b | (transform* adj (  (follicular adj/3 lymphoma) or  (chronic adj lymphocytic adj leuk?emia?) or  (small adj lymphocytic adj lymphoma?) or  richter? or  (nodular adj lymphocyte adj predominant adj/2 lymphoma?) or  (marginal adj zone adj/3 lymphoma?) or  (MALT? or (mucosa adj associated adj lymphoid adj tissue?))  )) |  |
| 3b | ((Waldenstr?m? adj macroglobulinemia?) or  (Richter? adj (syndrome? or lymphoma? or transformation?)) or  (Hodkins adj disease)) |  |
| 4b | ((mutli* adj myeloma?) or multi???myeloma? or  ((myelodysplastic adj syndrome?) or  myelodysplasia?) or  myelofibrosis or  (Hodkins adj disease?)) |  |
| 5b | 1b or 2b or 3b or 4b |  |
| 6 | 4a or 5b |  |
| 7 | ethnic* | *Group terms* |
| 8 | (race? or racial* or racism) |  |
| 9 | (multicultural or (multi adj cultural)) |  |
| 10 | (crosscultural or (cross adj cultural)) |  |
| 11 | (transcultural or (trans adj cultural)) |  |
| 12 | 7 or 8 or 9 or 10 or 11 |  |
| 13 | (bame? or black? or continental? or hispanic? or native? or oceanic? or asian? or indian? or pakistani or bangladeshi or chinese or african? or caribbean? or welsh or scottish or irish or gyps* or traveller? or roma or arab? or filipino? or indian? or showm?n or showwom?n or (show adj m?n) or (show adj wom?n)) |  |
| 14 | (population* or subpopulation* or subgroup* or group or ethnic or race* or adult* or age? or adolescent? or child* or infant? or person? or individual?) |  |
| 15 | 13 adj5 14 |  |
| 16 | 12 or 15 |  |
| 17 | 6 and 16 | *Disease terms* AND *group terms* |
| 18 | Limit 17 to English language and UK studies (custom limit used for UK studies where search tool limit not available^1^) | *UK terms* |
| 19 | 18 and ("car" or "cart" or "chimeric antigen receptor" or "chimeric antigen receptors" or "stem cell transplant" or "stemcell transplant" or "stem cells transplant" or "stemcells transplant" or "stem cell transplants" or "stemcell transplants" or "stem cells transplants" or "stemcells transplants" or "transplant stem" or "transplants stem" or "stem cell transplanted" or "stemcell transplanted" or "stem cells transplanted" or "stemcells transplanted" or "marrow transplant" or "marrows transplant" or "marrow transplants" or "marrows transplants" or "transplant marrow" or "transplants marrow" or "transplants marrows" or "marrow transplanted" or "marrows transplanted") | *Therapy terms* |
| * Evidence-Based Medicine Reviews (EBMR) [Internet]. [cited 2023 Nov 21]. Available from: https://www.wolterskluwer.com/en/solutions/ovid/evidencebased-medicine-reviews-ebmr-904  ^1^ UK or "United Kingdom*" or England* or English or Scotland* or Scottish or Wales or Welsh or Ireland* or Irish or Britain* or GB or British or NHS or "National Health Service" or "Bath and North East Somerset" or "Bedford" or "Bedford" or "Blackburn with Darwen" or "Blackpool" or "Bournemouth" or "Bracknell Forest" or "Sandhurst" or "Brighton and Hove" or "Brighton" or "Hove" or "Bristol" or "Buckinghamshire" or "Aylesbury Vale" or "Aylesbury" or "Chiltern" or "Amersham" or "Chalfont St. Giles" or "South Bucks" or "Beaconsfield" or "Stoke Poges" or "Wycombe" or "High Wycombe" or "Marlow" or "Cambridgeshire" or "East Cambridgeshire" or "Ely" or "Fenland" or "Wisbech" or "Huntingdonshire" or "Huntingdon" or "Ramsey" or "St. Ives" or "South Cambridgeshire" or "Cambridge" or "Central Bedfordshire" or "Ampthill" or "Dunstable" or "Cheshire East" or "Congleton" or "Crewe" or "Knutsford" or "Macclesfield" or "Nantwich" or "Cheshire West and Chester" or "Chester" or "Northwich" or "Cornwall" or "Bodmin" or "Falmouth" or "Fowey" or "Helston" or "Launceston" or "Looe" or "Lostwithiel" or "Newquay" or "Penryn" or "Penzance" or "St. Austell" or "Saltash" or "Tintagel" or "Truro" or "Cumbria" or "Allerdale" or "Cockermouth" or "Keswick" or "Workington" or "Barrow-in-Furness" or "Carlisle" or "Copeland" or "Whitehaven" or "Eden" or "Penrith" or "South Lakeland" or "Grasmere" or "Kendal" or "Darlington" or "Derby" or "Derbyshire" or "Amber Valley" or "Belper" or "Bolsover" or "Chesterfield" or "Derbyshire Dales" or "Ashbourne" or "Matlock" or "Erewash" or "High Peak" or "North East Derbyshire" or "South Derbyshire" or "Repton" or "Devon" or "East Devon" or "Axminster" or "Exmouth" or "Sidmouth" or "Exeter" or "Mid Devon" or "Crediton" or "North Devon" or "Barnstaple" or "Lynton and Lynmouth" or "South Hams" or "Dartmouth" or "Totnes" or "Teignbridge" or "Ashburton" or "Dawlish" or "Newton Abbot" or "Teignmouth" or "Torridge" or "Bideford" or "West Devon" or "Okehampton" or "Dorset" or "Christchurch" or "East Dorset" or "Wimborne Minster" or "North Dorset" or "Purbeck" or "Corfe Castle" or "West Dorset" or "Dorchester" or "Lyme Regis" or "Weymouth and Portland" or "Durham" or "Barnard Castle" or "Chester-le-Street" or "Durham" or "East Riding of Yorkshire" or "Beverley" or "Goole" or "East Sussex" or "Eastbourne" or "Hastings" or "Lewes" or "Lewes" or "Newhaven" or "Rother" or "Battle" or "Bexhill" or "Rye" or "Winchelsea" or "Wealden" or "Crowborough" or "Herstmonceux" or "Pevensey" or "Essex" or "Basildon" or "Braintree" or "Brentwood" or "Castle Point" or "Chelmsford" or "Colchester" or "Epping Forest" or "Chigwell" or "Harlow" or "Maldon" or "Burnham-on-Crouch" or "Rochford" or "Tendring" or "Harwich" or "Uttlesford" or "Saffron Walden" or "Gloucestershire" or "Cheltenham" or "Cotswold" or "Cirencester" or "Forest of Dean" or "Gloucester" or "Stroud" or "Tewkesbury" or "Tewkesbury" or "Winchcombe" or "Greater London" or "Camden" or "Bloomsbury" or "City of London" or "Smithfield" or "City of Westminster" or "Charing Cross" or "St. Marylebone" or "Soho" or "Hackney" or "Hammersmith and Fulham" or "Haringey" or "Islington" or "Clerkenwell" or "Kensington and Chelsea" or "Lambeth" or "Vauxhall" or "Lewisham" or "Newham" or "Southwark" or "Dulwich" or "Tower Hamlets" or "Limehouse" or "Wandsworth" or "Battersea" or "Barking and Dagenham" or "Barnet" or "Bexley" or "Brent" or "Bromley" or "Croydon" or "Ealing" or "Enfield" or "Greenwich" or "Woolwich" or "Harrow" or "Havering" or "Hillingdon" or "Hounslow" or "Kingston upon Thames" or "Merton" or "Wimbledon" or "Redbridge" or "Richmond upon Thames" or "Teddington" or "Sutton" or "Waltham Forest" or "Greater Manchester" or "Bolton" or "Bury" or "Manchester" or "Oldham" or "Rochdale" or "Salford" or "Stockport" or "Tameside" or "Trafford" or "Wigan" or "Atherton" or "Halton" or "Runcorn" or "Widnes" or "Hampshire" or "Basingstoke and Deane" or "Silchester" or "East Hampshire" or "Alton" or "Eastleigh" or "Fareham" or "Gosport" or "Hart" or "Havant" or "New Forest" or "Rushmoor" or "Test Valley" or "Andover" or "Romsey" or "Winchester" or "Hartlepool" or "Herefordshire" or "Hereford" or "Leominster" or "Ross-on-Wye" or "Hertfordshire" or "Broxbourne" or "Dacorum" or "Berkhamsted" or "Hemel Hempstead" or "East Hertfordshire" or "Bishop's Stortford" or "Hertford" or "Ware" or "Hertsmere" or "North Hertfordshire" or "Letchworth" or "St. Albans" or "Stevenage" or "Three Rivers" or "Watford" or "Welwyn Hatfield" or "Hatfield" or "Welwyn Garden City" or "Isle of Wight" or "Carisbrooke" or "Cowes" or "Freshwater" or "Newport" or "Ryde" or "Ventnor" or "Isles of Scilly" or "Hugh Town" or "Kent" or "Ashford" or "Canterbury" or "Herne Bay" or "Whitstable" or "Dartford" or "Dover" or "Deal" or "Dover" or "Sandwich" or "Gravesham" or "Gravesend" or "Maidstone" or "Sevenoaks" or "Edenbridge" or "Shepway" or "Folkestone" or "Hythe" or "Lydd" or "New Romney" or "Swale" or "Faversham" or "Thanet" or "Broadstairs and St. Peter's" or "Margate" or "Ramsgate" or "Tonbridge and Malling" or "Tunbridge Wells" or "Royal Tunbridge Wells" or "Kingston upon Hull" or "Lancashire" or "Burnley" or "Chorley" or "Fylde" or "Hyndburn" or "Lancaster" or "Pendle" or "Preston" or "Walton-le-Dale" or "Ribble Valley" or "Rossendale" or "South Ribble" or "West Lancashire" or "Skelmersdale" or "Wyre" or "Leicester" or "Leicestershire" or "Blaby" or "Charnwood" or "Loughborough" or "Harborough" or "Market Harborough" or "Hinckley and Bosworth" or "Melton" or "North West Leicestershire" or "Oadby and Wigston" or "Lincolnshire" or "Boston" or "East Lindsey" or "Lincoln" or "North Kesteven" or "South Kesteven" or "Grantham" or "Stamford" or "South Holland" or "Crowland" or "West Lindsey" or "Gainsborough" or "Luton" or "Medway" or "Chatham" or "Gillingham" or "Rochester" or "Merseyside" or "Knowsley" or "Huyton" or "Liverpool" or "St. Helens" or "Sefton" or "Southport" or "Wirral" or "Birkenhead" or "Middlesbrough" or "Milton Keynes" or "Norfolk" or "Breckland" or "East Dereham" or "Thetford" or "Broadland" or "Great Yarmouth" or "King's Lynn and West Norfolk" or "Castle Rising" or "King's Lynn" or "Sandringham" or "North Norfolk" or "Norwich" or "South Norfolk" or "North East Lincolnshire" or "Cleethorpes" or "Grimsby" or "North Lincolnshire" or "Scunthorpe" or "North Somerset" or "Weston-super-Mare" or "North Yorkshire" or "Craven" or "Hambleton" or "Northallerton" or "Harrogate" or "Knaresborough" or "Ripon" or "Richmondshire" or "Richmond" or "Ryedale" or "Malton" or "Scarborough" or "Whitby" or "Selby" or "Northamptonshire" or "Corby" or "Daventry" or "East Northamptonshire" or "Oundle" or "Kettering" or "Northampton" or "South Northamptonshire" or "Wellingborough" or "Northumberland" or "Bamburgh" or "Bedlington" or "Cramlington" or "Hexham" or "Morpeth" or "Warkworth" or "Nottingham" or "Nottinghamshire" or "Ashfield" or "Bassetlaw" or "Worksop" or "Broxtowe" or "Beeston and Stapleford" or "Gedling" or "Mansfield" or "Newark and Sherwood" or "Newark-on-Trent" or "Rushcliffe" or "West Bridgford" or "Oxfordshire" or "Cherwell" or "Banbury" or "Bicester" or "Oxford" or "South Oxfordshire" or "Henley-on-Thames" or "Vale of White Horse" or "Wantage" or "West Oxfordshire" or "Burford" or "Peterborough" or "Plymouth" or "Poole" or "Portsmouth" or "Reading" or "Redcar and Cleveland" or "Rutland" or "Uppingham" or "Shropshire" or "Bridgnorth" or "Ludlow" or "Much Wenlock" or "Oswestry" or "Shrewsbury" or "Stokesay" or "Slough" or "Somerset" or "Mendip" or "Glastonbury" or "Wells" or "Sedgemoor" or "Bridgwater" or "Cheddar" or "South Somerset" or "Ilchester" or "Langport" or "Taunton Deane" or "Taunton" or "Wellington" or "West Somerset" or "Dunster" or "Minehead" or "South Gloucestershire" or "Badminton" or "Kingswood" or "South Yorkshire" or "Barnsley" or "Doncaster" or "Adwick le Street" or "Rotherham" or "Sheffield" or "Southampton" or "Southend-on-Sea" or "Staffordshire" or "Cannock Chase" or "East Staffordshire" or "Burton upon Trent" or "Lichfield" or "Newcastle-under-Lyme" or "South Staffordshire" or "Stafford" or "Staffordshire Moorlands" or "Tamworth" or "Stockton-on-Tees" or "Stoke-on-Trent" or "Suffolk" or "Babergh" or "Sudbury" or "Forest Heath" or "Mildenhall" or "Newmarket" or "Ipswich" or "Mid Suffolk" or "St. Edmundsbury" or "Bury St. Edmunds" or "Suffolk Coastal" or "Dunwich" or "Felixstowe" or "Woodbridge" or "Waveney" or "Beccles" or "Lowestoft" or "Surrey" or "Elmbridge" or "Epsom and Ewell" or "Guildford" or "Mole Valley" or "Dorking" or "Reigate and Banstead" or "Runnymede" or "Spelthorne" or "Staines" or "Surrey Heath" or "Tandridge" or "Waverley" or "Haslemere" or "Woking" or "Swindon" or "Telford and Wrekin" or "Telford" or "Thurrock" or "Tilbury" or "Torbay" or "Brixham" or "Tyne and Wear" or "Gateshead" or "Felling" or "Newcastle upon Tyne" or "Newburn" or "North Tyneside" or "Wallsend" or "South Tyneside" or "Jarrow" or "South Shields" or "Sunderland" or "Washington" or "Warrington" or "Warwickshire" or "North Warwickshire" or "Nuneaton and Bedworth" or "Bedworth" or "Rugby" or "Stratford-on-Avon" or "Warwick" or "Royal Leamington Spa" or "Warwick" or "West Berkshire" or "Newbury" or "West Midlands" or "Birmingham" or "Coventry" or "Dudley" or "Sandwell" or "West Bromwich" or "Solihull" or "Walsall" or "Wolverhampton" or "West Sussex" or "Adur" or "Shoreham-by-Sea" or "Arun" or "Arundel" or "Bognor Regis" or "Chichester" or "Petworth" or "Crawley" or "Horsham" or "Mid Sussex" or "East Grinstead" or "Worthing" or "West Yorkshire" or "Bradford" or "Haworth" or "Keighley" or "Saltaire" or "Calderdale" or "Halifax" or "Todmorden" or "Kirklees" or "Dewsbury" or "Huddersfield" or "Leeds" or "Wakefield" or "Pontefract" or "Wiltshire" or "Amesbury" or "Bradford-on-Avon" or "Chippenham" or "Cricklade" or "Devizes" or "Malmesbury" or "Marlborough" or "Salisbury" or "Trowbridge" or "Westbury" or "Wilton" or "Windsor and Maidenhead" or "Ascot" or "Bray" or "Eton" or "Maidenhead" or "Windsor" or "Wokingham" or "Worcestershire" or "Bromsgrove" or "Malvern Hills" or "Great Malvern" or "Redditch" or "Worcester" or "Wychavon" or "Broadway" or "Droitwich" or "Evesham" or "Wyre Forest" or "Kidderminster" or "York" or "Antrim and Newtownabbey" or "Antrim" or "Newtownabbey" or "Ards and North Down" or "Newtownards" or "Bangor" or "Armagh, Banbridge, and Craigavon" or "Armagh" or "Banbridge" or "Dromore" or "Craigavon" or "Lurgan" or "Belfast" or "Stormont" or "Causeway Coast and Glens" or "Ballycastle" or "Ballymoney" or "Coleraine" or "Portrush" or "Limavady" or "Derry and Strabane" or "Londonderry" or "Strabane" or "Fermanagh and Omagh" or "Enniskillen" or "Omagh" or "Lisburn and Castlereagh" or "Lisburn" or "Mid and East Antrim" or "Ballymena" or "Carrickfergus" or "Larne" or "Mid Ulster" or "Cookstown" or "Dungannon" or "Magherafelt" or "Newry, Mourne, and Down" or "Downpatrick" or "Kilkeel" or "Newcastle" or "Newry" or "Aberdeen" or "Aberdeenshire" or "Banff" or "Braemar" or "Cruden Bay" or "Peterhead" or "St. Fergus" or "Angus" or "Arbroath" or "Brechin" or "Forfar" or "Glamis" or "Montrose" or "Argyll and Bute" or "Campbeltown" or "Dunoon" or "Inveraray" or "Lochgilphead" or "Rothesay" or "Tarbert" or "Clackmannanshire" or "Dumfries and Galloway" or "Dumfries" or "Gretna Green" or "Kirkcudbright" or "Lochmaben" or "Whithorn" or "Dundee" or "East Ayrshire" or "Cumnock" or "Kilmarnock" or "Mauchline" or "East Dunbartonshire" or "Kirkintilloch" or "Milngavie" or "East Lothian" or "Dunbar" or "Haddington" or "East Renfrewshire" or "Edinburgh" or "Leith" or "Falkirk" or "Falkirk" or "Grangemouth" or "Fife" or "Buckhaven" or "Culross" or "Cupar" or "Dunfermline" or "Glenrothes" or "Kirkcaldy" or "Rosyth" or "St. Andrews" or "Glasgow" or "Highland" or "Alness" or "Cawdor" or "Cromarty" or "Fort William" or "Invergordon" or "Inverness" or "John o'Groats" or "Nigg" or "Thurso" or "Wick" or "Inverclyde" or "Greenock" or "Midlothian" or "Dalkeith" or "Moray" or "Elgin" or "Forres" or "Lossiemouth" or "North Ayrshire" or "Irvine" or "North Lanarkshire" or "Coatbridge" or "Cumbernauld" or "Motherwell and Wishaw" or "Orkney Islands" or "Kirkwall" or "Perth and Kinross" or "Dunkeld" or "Kinross" or "Perth" or "Scone" or "Renfrewshire" or "Paisley" or "Renfrew" or "Scottish Borders" or "Coldstream" or "Duns" or "Galashiels" or "Hawick" or "Jedburgh" or "Kelso" or "Melrose" or "Newtown St. Boswells" or "Peebles" or "Selkirk" or "Shetland Islands" or "Lerwick" or "Sullom Voe" or "South Ayrshire" or "Ayr" or "Alloway" or "Prestwick" or "South Lanarkshire" or "East Kilbride" or "Hamilton" or "Lanark" or "Stirling" or "Balquhidder" or "Bannockburn" or "Callander" or "Stirling" or "West Dunbartonshire" or "Clydebank" or "Dumbarton" or "West Lothian" or "Linlithgow" or "Livingston" or "Western Isles" or "Stornoway" or "Blaenau Gwent" or "Abertillery" or "Ebbw Vale" or "Bridgend" or "Bridgend" or "Porthcawl" or "Caerphilly" or "Caerphilly" or "Gelligaer" or "Cardiff" or "Llandaff" or "Carmarthenshire" or "Carmarthen" or "Llanelli" or "Ceredigion" or "Aberystwyth" or "Cardigan" or "Conwy" or "Colwyn Bay" or "Conwy" or "Llandudno" or "Denbighshire" or "Denbigh" or "Rhyl" or "St. Asaph" or "Flintshire" or "Hawarden" or "Holywell" or "Gwynedd" or "Bala" or "Bangor" or "Caernarfon" or "Harlech" or "Isle of Anglesey" or "Holyhead" or "Llangefni" or "Merthyr Tydfil" or "Monmouthshire" or "Abergavenny" or "Chepstow" or "Monmouth" or "Usk" or "Neath Port Talbot" or "Margam" or "Neath" or "Pontardawe" or "Port Talbot" or "Newport" or "Caerleon" or "Pembrokeshire" or "Haverfordwest" or "Milford Haven" or "Pembroke" or "St. David's" or "Tenby" or "Powys" or "Brecon" or "Builth Wells" or "Llandrindod Wells" or "Montgomery" or "Newtown" or "Welshpool" or "Rhondda Cynon Taf" or "Aberdare" or "Hirwaun" or "Llantrisant" or "Mountain Ash" or "Pontypridd" or "Swansea" or "Swansea" or "Torfaen" or "Cwmbrân" or "Pontypool" or "Vale of Glamorgan" or "Barry" or "Cowbridge" or "Llantwit Major" or "Wrexham" or "Wrexham" or "Macmillan Cancer Support" or "Action Cancer" or "Cancer Card" or "CancerVoices" or "Maggies Centres" or "Marie Curie" or "National Cancer Institute" or "Penny Brohn UK" or "Bristol Cancer Help Centre" or "Cancer Support Scotland" or "Tak Tent" or "Tenovus Cancer Care" or "Cancer Focus Northern Ireland" or "Future Fertility Programme Oxford" or "HealthTalk"  ^1^ based on: Ayiku L, Levay P, Hudson T, Finnegan A. The NICE UK geographic search filters for MEDLINE and Embase (Ovid): Post-development study to further evaluate precision and number-needed-to-read when retrieving UK evidence. Research Synthesis Methods. 2020;11(5):669–77. | | |
