# Supplementary material for: Protocol: what are the ethnic inequities in care outcomes related to haematological malignancies, treated with transplant/cellular therapies, in the UK? A systematic review
Source: BMJ Open. 2025 May 21;15(5):e099354. doi: 10.1136/bmjopen-2025-099354 (PMC12096984; doi:10.1136/bmjopen-2025-099354)
Supplement: Supplementary file 3 [file bmjopen-2025-099354.reviewer_comments.pdf]

PEER REVIEW HISTORY

BMJ Open publishes all reviews undertaken for accepted manuscripts. Reviewers are asked to complete a checklist review form and are provided with free text boxes to elaborate on their assessment. These free text comments are reproduced below.

ARTICLE DETAILS

Title (Provisional)

Protocol: What are the ethnic inequities in care outcomes related to haematological malignancies, treated with transplant/cellular-therapies, in the UK? A Systematic Review.

Authors

Cusworth, Samuel; Deplano, Zareen; Denniston, Alastair K; Burns, David; Nirantharakumar, Krishnarajah; Adderley, Nicola; Chandan, Joht Singh

---

VERSION 1 - REVIEW

**Reviewer**	**1**
**Name**	**Shimadu, Yutaka**
**Affiliation**	**Osaka University, Experimantal Immunology**
**Date**	**05-Feb-2025**
**COI**	**None**

The authors clearly highlight ethnic inequities in care outcomes related to hematological malignancies in the UK. The manuscript is well-written; however, I have concerns regarding the formulation of the clinical question.

This study addresses disparities in the access to and outcomes of transplant/cellular therapies; however, the challenges associated with CAR-T therapy and hematopoietic stem cell transplantation (HSCT) differ in important ways.

For instance, CAR-T therapy is primarily limited by its high cost and restricted number of treatment centers, creating economic and geographic barriers. In contrast, HSCT is also affected by financial constraints; allogeneic HSCT presents an additional barrier in terms of donor availability, whereas autologous HSCT does not face this issue.

Furthermore, economic barriers are not unique to transplant/cellular therapies but are also relevant to novel drug therapies.

Given these distinct challenges, I questioned whether it is appropriate to assess these treatment modalities under a single clinical question. Without addressing this issue, meaningful discussion is difficult.

**Reviewer**	**2**
**Name**	**Shimomura, Yoshimitsu**
**Affiliation**	**Department of Hematology, Kobe City Medical Center General Hospital, Kobe, Japan**
**Date**	**13-Feb-2025**
**COI**	**None**

This study addresses an important topic. As a hematologist, I find the subject clinically relevant, and I look forward to seeing the results of this systematic review. Although I am not an expert in systematic reviews or meta-analyses, nor am I deeply familiar with the specifics of the UK healthcare system, I offer the following two comments from the perspective of a clinical hematologist. I hope they will prove helpful in strengthening the protocol.

Comment #1

At present, the manuscript groups chimeric antigen receptor (CAR) T-cell therapy and stem cell transplantation together. From a hematological standpoint, these are distinct treatment modalities with different indications and patient populations. For example, CART therapy is indicated for certain types of lymphoma and multiple myeloma. Auto-SCT is indicated for malignant lymphoma, multiple myeloma, and a part of leukemia. Allo-SCT had additional complexity regarding donor selection, particularly in the context of HLA compatibility. Each treatment modality might face its own set of barriers, inequities, and outcome determinants, which could be obscured. Therefore, it may be more appropriate to separate.

Comment #2

I recommend reviewing and possibly restructuring the classification and terminology used for hematological malignancies. A recognized classification system such as the "WHO Classification of Tumours of Haematopoietic and Lymphoid Tissues" can provide a solid framework. This would help ensure consistency and accuracy in describing disease entities. In the current text, some terms appear to be imprecise. For example, the sentence "the location and morphology, with the broad classifications of leukaemias (leukocytes), myelomas (myeloid cells) and lymphomas (lymphocytes)" on page 3 may be wrong. Myelomas are not myeloid cells. I recommended reviewing and revising the classification of hematological malignancies on Pages 6-7. By aligning the review's inclusion/exclusion criteria and disease definitions with a well-established classification system, you will improve clarity and reduce ambiguity regarding which subtypes of hematological malignancies are included.

**Reviewer**	**3**
**Name**	**Montoto, Silvia**

Affiliation St Bartholomew's Hospital

Date 17-Feb-2025

COI None

This protocol addresses a very important question with significant implications in terms of public health interventions and resources. The methodology that will be followed to address this question is extremely well detailed to ensure that the review is as complete and free of bias as possible.

Reviewer 4

Name Kuwatsuka, Yachiyo

Affiliation Nagoya University Hospital, Center for Advanced Medicine and Clinical Research

Date 18-Feb-2025

COI None

This protocol describes a systematic review on UK minority ethnic populations receiving haematopoetic stem cell transplantation or CAR-T therapy. I have several suggestions for the authors:

Methods

Search criteria include CAR-T therapy and stem cell transplantation. HLA as a genetic construct of race directly influences the allogeneic transplant outcomes, including survival. International registry studies have already provided insight into the effect of race/ethnicity on various transplant outcomes. In the context of race/ethnicity outcome studies, allogeneic transplantation is unique because HLA has a direct impact on outcomes and allogeneic transplantation could significantly bias the outcomes when combined with autologous or CAR-T when assessing the effect of race/ethnicity. I would like to strongly recommend performing subgroup analysis for CAR-T, autologous stem cell- and allogeneic stem cell-transplant for meta-analysis and to noting this in the data synthesis section. I would think that a narrative analysis to describe the treatment/care types or outcomes of each race/ethnicity group would not be a problem.

PRISMA-P check list

The line numbers in the submitted manuscript were not identifiable for the reviewer. It would be helpful if PRISMA line numbers could be identified in the manuscript for review.

VERSION 1 - AUTHOR RESPONSE

Reviewer: 1
Dr. Yutaka Shimadu, Osaka University
Comments to the Author:
The authors clearly highlight ethnic inequities in care outcomes related to hematological malignancies in the UK. The manuscript is well-written; however, I have concerns regarding the formulation of the clinical question.
This study addresses disparities in the access to and outcomes of transplant/cellular therapies; however, the challenges associated with CAR-T therapy and hematopoietic stem cell transplantation (HSCT) differ in important ways.
For instance, CAR-T therapy is primarily limited by its high cost and restricted number of treatment centers, creating economic and geographic barriers. In contrast, HSCT is also affected by financial constraints; allogeneic HSCT presents an additional barrier in terms of donor availability, whereas autologous HSCT does not face this issue.
Furthermore, economic barriers are not unique to transplant/cellular therapies but are also relevant to novel drug therapies.
Given these distinct challenges, I questioned whether it is appropriate to assess these treatment modalities under a single clinical question. Without addressing this issue, meaningful discussion is difficult.

Reply:

Thank you for the response, and for taking the time to review the protocol. We greatly appreciate your advice and feedback.

We agree that the above barriers to treatments have different effects on the different treatments, but we are focussing primarily on the mortality statistics, with some factors that could impact these (stage of cancer at detection/referral, treatments/interventions received, methods of diagnosis, time intervals between care pathway stages). We are specifically looking at ethnic inequalities, and feel the above outcomes are (1) possible to extract from quantitative studies, (2) will be relevant across both CAR-T and HSCT.

We want to provide an overview of the general evidence in this area, that will help decide where to focus future research (e.g. in some of the more specific areas explained in your comments).

From piloting, we expect a very small pool of evidence to analyse, therefore we wanted to keep the focus broad.

However, if we do find sufficient literature, we will also undertake an enhanced narrative review to discuss the outcomes by treatment (i.e. HSCT and CAR-T), to ensure the results are discussed in the clearest, and most meaningful way.

Reviewer: 2
Dr. Yoshimitsu Shimomura, Department of Hematology, Kobe City Medical Center General Hospital, Kobe, Japan
Comments to the Author:
This study addresses an important topic. As a hematologist, I find the subject clinically relevant, and I look forward to seeing the results of this systematic review. Although I am not an expert in systematic reviews or meta-analyses, nor am I deeply familiar with the specifics of the UK healthcare system, I offer the following two comments from the perspective of a clinical hematologist. I hope they will prove helpful in strengthening the protocol.

Comment #1
At present, the manuscript groups chimeric antigen receptor (CAR) T-cell therapy and stem cell transplantation together. From a hematological standpoint, these are distinct treatment modalities with different indications and patient populations. For example, CART therapy is indicated for certain types of lymphoma and multiple myeloma. Auto-SCT is indicated for malignant lymphoma, multiple myeloma, and a part of leukemia. Allo-SCT had additional complexity regarding donor selection, particularly in the context of HLA compatibility. Each treatment modality might face its own set of barriers, inequities, and outcome determinants, which could be obscured. Therefore, it may be more appropriate to separate.

Comment #2
I recommend reviewing and possibly restructuring the classification and terminology used for hematological malignancies. A recognized classification system such as the "WHO Classification of Tumours of Haematopoietic and Lymphoid Tissues" can provide a solid framework. This would help ensure consistency and accuracy in describing disease entities. In the current text, some terms appear to be imprecise. For example, the sentence "the location and morphology, with the broad classifications of leukaemias (leukocytes), myelomas (myeloid cells) and lymphomas (lymphocytes)" on page 3 may be wrong. Myelomas are not myeloid cells. I recommended reviewing and revising the classification of hematological malignancies on Pages 6-7. By aligning the review's inclusion/exclusion criteria and disease definitions with a well-established classification system, you will improve clarity and reduce ambiguity regarding which subtypes of hematological malignancies are included.

Reply:

Thank you for taking the time to read the protocol and providing feedback. We really do appreciate your feedback.

Comment 1: We thank the reviewer and also reviewer 1 who raised this. Hence, we will amend the protocol to group outcomes by treatment (i.e. HSCT and CAR-T), to ensure the results are discussed in the clearest, and most meaningful way.

Comment 2:

We are extremely grateful for the comment. We sought advice from local UK clinical haematology experts, and their recommendation was to use BSBMTCT (spell out) classification due to relevance to UK haematological malignancies.

Thank you for highlighting this. We have changed that section to the following: 'They cover a variety of different cancers, diagnosed by the cells effected, the location and morphology, with the broad classifications of leukaemias (early phase blood cells in bone marrow and/or blood), myelomas (plasma cells in bone marrow) and lymphomas (lymphoid cells in lymphatics).'

We have also removed the list of transformed lymphomas (was unclear whether listed were lymphomas that could transform, or those that had transformed), and added any relevant key conditions to the other lists.

Reviewer: 3
Dr. Silvia Montoto, St Bartholomew's Hospital
Comments to the Author:
This protocol addresses a very important question with significant implications in terms of public health interventions and resources. The methodology that will be followed to address this question is extremely well detailed to ensure that the review is as complete and free of bias as possible.

Reply:

Thank you for reading the protocol and providing supportive comments.

Reviewer: 4
Dr. Yachiyo Kuwatsuka, Nagoya University Hospital
Comments to the Author:
This protocol describes a systematic review on UK minority ethnic populations receiving haematopoetic stem cell transplantation or CAR-T therapy. I have several suggestions for the authors:

Methods

Search criteria include CAR-T therapy and stem cell transplantation. HLA as a genetic construct of race directly influences the allogeneic transplant outcomes, including survival. International registry studies have already provided insight into the effect of race/ethnicity on various transplant outcomes. In the context of race/ethnicity outcome studies, allogeneic transplantation is unique because HLA has a direct impact on outcomes and allogeneic transplantation could significantly bias the outcomes when combined with autologous or CAR-T when assessing the effect of race/ethnicity. I would like to strongly recommend performing subgroup analysis for CAR-T, autologous stem cell- and allogeneic stem cell-transplant for meta-analysis and to noting this in the data synthesis section. I would think that a narrative analysis to describe the treatment/care types or outcomes of each race/ethnicity group would not be a problem.

Reply:

Thank you so much for your kind review, we really appreciate it. Also we are in agreement as with reviewer 1 and 2 and hence, amended the protocol to group the narrative synthesis and meta-analysis by treatment (autologous HSCT vs allogeneic HSCT, vs CAR-T).
